# Phytochemical screening*,* antimycobacterial activity and acute toxicity of crude extracts of selected medicinal plant species used locally in the treatment of tuberculosis in Uganda

**DOI:** 10.1186/s41182-022-00406-7

**Published:** 2022-02-17

**Authors:** Benson Oloya, Jane Namukobe, Willy Ssengooba, Mathias Afayoa, Robert Byamukama

**Affiliations:** 1 Department of Chemistry, College of Natural Sciences, Makerere University, P.O. Box 7062, Kampala, Uganda; 2 Department of Chemistry, Faculty of Science, Muni University, P.O. Box 725, Arua, Uganda; 3 Department of Medical Microbiology, College of Health Science, Makerere University, P.O. Box 7072, Kampala, Uganda; 4 Department of Pharmacy, Clinical and Comparative Veterinary Medicine, College of Veterinary Medicine, Animal Resources and Biosecurity, Makerere University, P.O. Box 7072, Kampala, Uganda

**Keywords:** Phytochemistry, Antimycobacterial activity, Acute toxicity, Tuberculosis, Medicinal plants

## Abstract

**Background:**

Tuberculosis (TB) is one of the leading causes of death globally, and the rise in drug-resistant forms of TB has become a significant threat. Subsequently, it is crucial to explore new, effective and safe anti-TB agents. This study aimed at conducting phytochemical screening, antimycobacterial activity, and acute toxicity of the selected plant species’ crude extracts to assess their toxicological potentials and efficacies against TB.

**Methods:**

The aqueous and methanol/dichloromethane (DCM) (1:1) extracts of each selected plant species were subjected to phytochemical screening and antimycobacterial activity using microplate alamar blue assay. For acute toxicity, a single dose (2000 mg/kg) of the aqueous extracts was orally administered to each animal following the Organization for Economic Cooperation and Development (OECD) guidelines No. 425 and then observed for 14 days. The animals were closely observed on the general behavior and clinical signs of toxicity, and body weights were recorded. After the termination of the experiment, hematological, biochemical, and histopathological analyses were performed.

**Results:**

The extracts contained alkaloids, flavonoids, tannins, saponins, steroids, terpenoids, resins, cardiac glycosides, phenolic compounds, and coumarins. Aqueous extracts showed moderate to weak activity against the susceptible (H_37_Rv) *M. tuberculosis* strain and weak activity against the MDR-TB strain with Minimum Inhibitory Concentrations (MIC μg/mL) ranging from 293.0–2344.0 and 1172.0–4688.0, respectively. Methanol/DCM extracts showed significant to moderate activity against the susceptible TB strain and moderate to weak activity against the MDR-TB strain with MIC (μg/mL) ranging from 98.0–586.0 and 293.0–781.0, respectively. One mortality was recorded from the *A. coriaria* treated group following the acute toxicity tests, but the LD_50_ of all the extracts was estimated to be above 2000 mg/kg. Histopathological analyses did not show any significant lesions in the examined organs except those from the *A. coriaria* treated group.

**Conclusion:**

Phytochemical screening of the extracts revealed the presence of alkaloids, tannins, saponins, flavonoids, steroids, terpenoids, resins, cardiac glycosides, phenolic compounds, and coumarins. All the methanol/DCM extracts of the plant species studied have promising antimycobacterial activity. The selected plant extracts studied exhibited low acute toxicity levels except for *A. coriaria* and could be safe for formulations into herbal products.

**Supplementary Information:**

The online version contains supplementary material available at 10.1186/s41182-022-00406-7.

## Background

Tuberculosis (TB) has been ranked among the top causes of death worldwide and the leading cause of death emanating from a single infectious agent, and since 2007, ranks above HIV/AIDS [1]. The emergence of drug-resistant TB has exacerbated this problem. There is a need for multifaceted interventions to reverse this trend. One viable option is exploring more effective treatments derived from medicinal plants because from the earliest days of humanity, they have been the foundation of health care globally. In addition, medicinal plants have contributed to modern drug discovery and are still being used widely with significant importance in international trade [2, 3]. About 90% of the population in some countries in Africa still depend exclusively on medicinal plant products as sources of medicines [4].

Furthermore, scientific attention to medicinal plants has significantly increased recently. This resurgence mainly arises due to the increased efficacy of novel drugs of plant origin, growing fears about the after-effects of contemporary medicine, and the emergence of resistance to existing drugs. Therefore, it is indispensable to search for novel molecular structures as templates for drug development from plants [5]. From history, the most outstanding sources of new anti-TB drugs have been natural products, although there are other potential sources, such as synthetic and combinatorial chemistry [6]. Moreover, some studies have shown that plants have antimycobacterial activity and contain antimycobacterial compounds [7–10]. From ethnopharmacological surveys, the leaves, stem, or root barks of the following medicinal plant species, selected for investigation in this study, have been used to manage TB by the local people in Uganda: *Acacia hockii*, *Albizia coriaria*, *Combretum molle, Warburgia ugandensis*, and *Zanthoxylum leprieurii* [11–15].

Medicinal plants often possess various phytoconstituents, many of them with unidentified biological properties, which could be toxic and cause drug interactions that are harmful to human health [16]. Consequently, assessment of the toxicity of a substance is critical when considering it for use as a drug since exposure to toxic substances is dangerous to both humans and animals [17]. Besides, there is limited documentation on the safety profile of plant-derived medicines, which does not satisfy the regulatory criteria of the category of drugs. It is necessary to undertake systematic studies of natural products and their phytoconstituents regarding their effectiveness and safety before using them as botanical drugs [18, 19].

This study focused on analyzing the methanol/DCM (1:1, v/v) and aqueous crude extracts of the selected plant species to assess their toxicological potentials and efficacies against *M. tuberculosis*.

## Materials and methods

### Selection criteria

The medicinal plant species considered in the present study were selected based on their high frequency of mention registered from three or more different ethnobotanical surveys reported in the literature for the treatment of TB. In addition, they had no reports of similar studies using the solvent systems employed in the present study.

### Plant material

Plant parts from *Acacia hockii* De Wild. (Fabaceae), *Albizia coriaria* Oliv. (Fabaceae)*,* and *Combretum molle* R.Br. ex G.Don (Combretaceae) were collected from Zombo district, Uganda, while the stem bark of *Warburgia ugandensis* Sprague (Canellaceae) was harvested from Mabira Forest in Buikwe district, Uganda, and the root bark of *Zanthoxylum leprieurii* Guill. & Perr. (Rutaceae) was from Mpanga Forest Reserve in Mpigi district, Uganda, in November 2020. Botanical identification was done by a taxonomist from Makerere University Herbarium and voucher specimen Nos.: O.B. 15, Accession No. 50967 (*Zanthoxylum leprieurii*); O.B. 16, Accession No. 50999 (*Warburgia ugandensis* Sprague); O.B. 17, Accession No. 51000 (*Combretum molle*); O.B. 18, Accession No. 51001 (*Acacia hockii*); O.B. 19, Accession No. 51002 (*Albizia coriaria*) were deposited at Makerere University Herbarium. The plant parts were air-dried at room temperature for at least 3 weeks and then pounded into fine powder.

### Extraction process

For organic extractions, the powdered plant part (60 g) of each of the following: *A. hockii* (stem bark), *A. coriaria* (stem bark), *C. molle* (leaves), *W. ugandensis* (stem bark), and *Z. leprieurii* (root bark) was soaked in a mixture of methanol/DCM (1:1, v/v) (400 mL) for 24 h, with occasional shaking. The extracts were filtered twice: first, with cotton wool, and second, with a filter paper (Whatman No. 1). After that, the filtrates were separately concentrated to dryness using a Rotary Evaporator running at 35–40 °C and under reduced pressure. The extracts obtained from *A. hockii* (6.71 g), *A. coriaria* (8.10 g), *C. molle* (4.05 g), *W. ugandensis* (6.50 g), and *Z. leprieurii* (5.22 g) were stored at − 4 °C awaiting phytochemical screening, and antimycobacterial activity testing.

For aqueous extractions, the powdered plant part (200 g) of each of the following: *A. hockii* (stem bark), *A. coriaria* (stem bark), *C. molle* (leaves), *W. ugandensis* (stem bark), and *Z. leprieurii* (root bark) was soaked in hot distilled water (1200 mL) at 60 °C and shaken well. The mixtures were heated in a water bath kept at 60 °C for 2 h with frequent shaking. The extracts were subjected to a triple filtration: first, with a cotton piece of cloth, and then second and third, with cotton wool. The filtrates were subjected to freeze-drying, and the extracts obtained from *A. hockii* (12.84 g), *A. coriaria* (10.93 g), *C. molle* (17.50 g), *W. ugandensis* (15.51 g), and *Z. leprieurii* (11.85 g), were stored at − 4 °C awaiting phytochemical screening, antimycobacterial activity and acute toxicity testing.

### Phytochemical screening of extracts

The methanol/DCM (1:1) and aqueous extracts from each selected plant species were analyzed for alkaloids, saponins, tannins, flavonoids, steroids, cardiac glycosides, resins, anthraquinones, phenolic compounds, phlobatannins, coumarins, and terpenoids. Standard methods for preliminary phytochemical analysis were used with some minor modifications [20, 21]. A detailed description of the method has been included in the Additional file 1.

### Antimycobacterial activity

#### M. tuberculosis strains and preparation of inoculum

Two experimental mycobacterial strains from a WHO proficiency testing panel were used: a fully susceptible laboratory strain (H_37_Rv) and a known MDR-TB (375) strain. The strains used were obtained from the Mycobacteriology Laboratory (BSL-3) at the College of Health Sciences, Makerere University. This laboratory has been accredited by the College of American Pathologists (CAP) ISO 15189.

The preserved mycobacterial strains were cultured on Middlebrook 7H10 agar before the susceptibility tests [22, 23]. Cells that were scraped from freshly growing colonies (3 weeks old) were introduced into normal saline (10 mL). A bacterial suspension equivalent to 0.5 McFarland standards (1.5 × 10^8^ CFU) was prepared using a Sensititre Nephelometer by adding more cells or diluting with more normal saline [23, 24].

#### Preparation of plant extracts

The dried plant extracts (1 g) were dissolved separately in DMSO (10 mL) to give a concentration of 100 mg/mL and later sterilized using 0.2 μm single-use filters before use.

#### Determination of minimum inhibitory concentration (MIC) and minimum bactericidal concentration (MBC)

The MIC and MBC of the crude extracts against both the susceptible (H_37_Rv) and MDR-TB strains were determined using microplate alamar blue assay (MABA) protocol with minor modifications [25]. A detailed description has been included in the Additional file 1.

### Experimental animals

Adult female Wistar albino rats (aged 8–10 weeks) were used for the acute toxicity study. Female rats were preferred, since earlier studies have suggested they are generally more sensitive to toxins than male rats [26]. The animals were fed on pellets (standard diet) obtained from Nuvita Animal Feeds (Jinja, Uganda). The animals had access to tap water *ad libitum* and were kept at standard laboratory conditions of ventilation, regular 12 h light/12 h dark cycle, and temperature (20–30 °C) during the experimental period. The animals were acclimatized to standard laboratory conditions (in plastic cages with stainless steel top) for 2 weeks before the experiments.

### Acute toxicity assay

Acute toxicity study was performed in line with the OECD standard guidelines for using animals in scientific research, Guideline No. 425 [27], with minor modifications. Thirty rats were allocated randomly into six groups, each with five rats as follows according to the plant extract administered: Group I (*Z. leprieurii*-root bark), II (*W. ugandensis*-stem bark), III (*C. molle*-leaves), IV (*A. coriaria*-stem bark), V (*A. hockii*-stem bark) and VI (Control). After a 24 h starvation (no food provided except water), the single fixed dose of 2000 mg/kg of the aqueous extract was administered to each rat from Groups I to V by gavage using an endogastric tube. Rats from Group VI received distilled water (2 mL) only. The animals were observed regularly and individually for the first 24 h after dosing for behavioral and general toxicity signs, and particular attention was given during the first 4 h. After that, the animals were given unlimited access to water and food, and observation continued daily for 14 days on clinical signs of toxicity, mortality, and general behavior. Body weights of the animals were measured on days: 0, 7, 10, and 14. After the termination of the experiment, three rats from each group were starved overnight and later anesthetized with chloroform. When the anesthesia had reached depth, a cardiac puncture was performed on each of the three selected rats per group, and blood samples were collected for biochemical and hematological analyses. Afterward, the animals were euthanized by cervical dislocation, and the following vital organs: heart, kidney, and liver, were removed and processed for histopathological analysis.

### Hematological analysis

Blood samples were stored in EDTA tubes, and hematological analysis was performed using an automated hematology analyzer (SYSMEX XN-L 450 SN 11097). The main parameters evaluated included red blood cells, hemoglobin, mean corpuscular volume, mean corpuscular hemoglobin, mean corpuscular hemoglobin concentration, hematocrit, platelets, white blood cells, lymphocytes, monocytes, and neutrophils.

### Biochemical analysis

Dry tubes containing the collected blood samples were centrifuged at 3000 rpm for 15 min to obtain the serum. An automated analyzer (COBAS 6000) was used to evaluate the following parameters: aspartate aminotransferase, alanine aminotransferase, total serum protein, bilirubin, alkaline phosphatase, albumin, serum creatinine, and serum urea [28].

### Histopathological analysis

The vital organs collected from the animals were washed with saline solution 0.9% (w/v), weighed, and then fixed in 10% buffered formaldehyde–calcium solution at a ratio of 1:10 (v/v). The organs were trimmed and processed in an automated tissue processor (Leica TP 1020, German) for paraffin embedding. Sections (5 µm thick) were prepared, adhered onto microscope slides, and stained with hematoxylin and eosin (HE). The tissue sections were examined under a microscope (Nikon Eclipse Phase Contrast, Japan) for their general structure, lesions associated with toxicity such as degenerative changes, necrosis, and evidence of inflammatory response were recorded and described. The images of the various tissue sections and lesions were captured using a microphotographic imaging system (Nikon digital Sight DS-F 11) [29].

### Statistical analysis

The relative weight of organs, biochemical, and hematological data were expressed as mean ± standard error of the mean (SEM). The data were subjected to One-Way ANOVA followed by Dunnett’s comparison test to compare each group with the Control. A *p* value ˂ 0.05 was considered statistically significant. The statistical analysis was carried out using SPSS (IBM SPSS Statistics 20).

## Results

### Phytochemical constituents

The extracts contained alkaloids, saponins, tannins, flavonoids, steroids, resins, terpenoids, cardiac glycosides, phenolic compounds, and coumarins (Table 1).

**Table 1:** Phytochemical constituents of both the aqueous and methanol/DCM (1:1) extracts

Phytochemical compounds	Plant species									
	AH-SB		AC-SB		CML		WU-SB		ZL-RB	
	AE	OE	AE	OE	AE	OE	AE	OE	AE	OE
Alkaloids	+	+	+	+	+	+	−	+	+	+
Saponins	+	+	+	+	−	−	+	+	+	−
Tannins	+	+	+	+	+	+	+	+	+	−
Flavonoids	+	+	−	−	+	+	−	−	−	−
Steroids	−	−	−	+	+	−	+	+	+	+
Cardiac glycosides	−	−	−	+	+	+	+	+	+	+
Resins	−	−	−	−	+	+	−	+	+	+
Anthraquinones	−	−	−	−	−	−	−	−	−	−
Phenolic compounds	+	+	+	+	+	+	+	−	+	+
Phlobatannins	−	−	−	−	−	−	−	−	−	−
Coumarins	−	−	−	+	+	+	+	+	+	+
Terpenoids	+	+	+	+	+	+	+	+	+	+

*AH-SB* *Acacia hockii* stem bark, *AC-SB* *Albizia coriaria* stem bark, *CM-L* *Combretum molle* leaves, *WU-SB* *Warburgia ugandensis* stem bark, *ZL-RB* *Zanthoxylum leprieurii* root bark, *AE* aqueous extract, *OE* Organic extract [methanol/DCM (1:1)]; (+) = present; (−) = absent

### Antimycobacterial activity

The MIC and MBC values for both the susceptible H_37_Rv and MDR-TB strains for aqueous and methanol/DCM (1:1) extracts are shown in Tables 2 and 3, respectively. For aqueous extracts (Table 2), *A. hockii* (stem bark) showed a moderate antimycobacterial activity (MIC, 293 μg/mL) against the susceptible H_37_Rv strain but a low activity (MIC, 1172 μg/mL) against the MDR-TB strain. The other species: *A. coriaria*, *C. molle*, *W. ugandensis*, and *Z. leprieurii* (root bark), showed low activities against both the susceptible H_37_Rv and MDR-TB strains.

**Table 2:** Mean MIC and MBC values of aqueous crude extracts of selected plant species on susceptible and MDR strains of *M. tuberculosis*

Plant species-part	Mean MIC (μg/mL)		Mean MBC (μg/mL)	
	H_37_Rv	MDR	H_37_Rv	MDR
AH-SB	293.00 ± 138.60	1172.00 ± 553.00	391.00 ± 0.00	781.00 ± 0.00
AC-SB	2344.00 ± 1105.00	4688.00 ± 2210.00	1563.00 ± 0.00	4688.00 ± 2210.00
CM-L	1172.00 ± 553.00	2344.00 ± 11.05.00	1563.00 ± 0.00	1172.00 ± 553.00
WU-SB	1172.00 ± 553.00	1563.00 ± 0.00	2344.00 ± 1105.00	1563.00 ± 0.00
ZL-RB	781.00 ± 0.00	1563.00 ± 0.00	2344.00 ± 1105.00	2344.00 ± 1105.00

*AH-SB* *Acacia hockii* stem bark, *AC-SB* *Albizia coriaria* stem bark, *CM-L* *Combretum molle* leaves, *WU-SB* *Warburgia ugandensis* stem bark, *ZL-RB* *Zanthoxylum leprieurii* root bark. Values are presented as mean ± Standard deviation

**Table 3:** Mean MIC and MBC values of methanol/DCM (1:1) crude extracts of different plant species obtained from different strains of *M. tuberculosis*

Plant species-part	Mean MIC (μg/mL)		Mean MBC (μg/mL)	
	H_37_Rv	MDR	H_37_Rv	MDR
AH-SB	98.00 ± 0.00	293.00 ± 138.60	391.00 ± 0.00	781.00 ± 0.00
AC-SB	391.00 ± 0.00	781.00 ± 0.00	781.00 ± 0.00	2344.00 ± 1105.00
CM-L	293.00 ± 138.60	391.00 ± 0.00	781.00 ± 0.00	1563.00 ± 0.00
WU-SB	586.00 ± 275.80	586.00 ± 275.80	1563.00 ± 0.00	2344.00 ± 1105.00
ZL-RB	195.00 ± 0.00	293.00 ± 138.6	3125.00 ± 0.00	2344.00 ± 1105.00

*AH-SB* *Acacia hockii* stem bark, *AC-SB* *Albizia coriaria* stem bark, *CM-L* *Combretum molle* leaves, *WU-SB* *Warburgia ugandensis* stem bark, *ZL-RB* *Zanthoxylum leprieurii* root bark. Values are presented as mean ± Standard deviation

For the methanol/DCM (1:1) extracts (Table 3), the five plant extracts had significant to moderate activities against the susceptible H_37_Rv and moderate to low activities against the MDR-TB strain. In particular, *A. hockii* showed a significant antimycobacterial activity (MIC, 98 μg/mL) against the susceptible H_37_Rv but a moderate activity against the MDR-TB strain (MIC, 293 μg/mL)*.* Also, *Z. leprieurii*, *C. molle*, and *W. ugandensis* showed moderate activity against the susceptible H_37_Rv and MDR-TB strains. In addition, *A. coriaria* showed moderate activity against the susceptible H_37_Rv strain but a low activity against the MDR-TB strain.

### Acute toxicity

#### General signs and mortality

The parameters observed for acute toxicity signs after administering the plant extracts are shown in Table 4. No significant changes in behavior were observed in the treated groups following the acute toxicity study. However, one death was recorded from the *A. coriaria* (stem bark) treated group on the fourth day during the 14 days of the experiment. In addition, there was lethargy in all the treated groups for up to 4 h following administration of the extracts. Increased respiration was observed in the *A. coriaria* and *Z. leprieurii* (root bark) treated groups. There was excessive urination and increased defecation in the *A. coriaria* (stem bark) treated group for 3 days after administration. Furthermore, there was excessive urination in the *A. hockii* (stem bark) treated group for up to 2 days after administration.

**Table 4:** General appearance and behavioral patterns of animals from the treated and Control group

Parameters	Plant species						
	Ctrl	AH-SB	AC-SB	CM-L	WU-SB	ZL-RB	
Mortality	0/5	0/5	1/5	0/5	0/5	0/5	
Urination (excessive)	x	√	√	x	x	x	
Defecation	N	N	↑	N	N	N	
Diarrhea	x	x	x	x	x	x	
Salivation	x	x	x	x	x	x	
Respiration (within 4 h)	N	N	↑	N	N	↑	
Sleep	N	N	N	N	N	N	
Itching	x	x	x	x	x	x	
Convulsions/tremors	x	x	x	x	x	x	
Lethargy (within 4 h)	x	√	√	√	√	√	
Coma	x	x	x	x	x	x	

N-normal, x-not observed, ↑-increased, √-observed

#### Body and relative organ weights

**Fig. 1:**
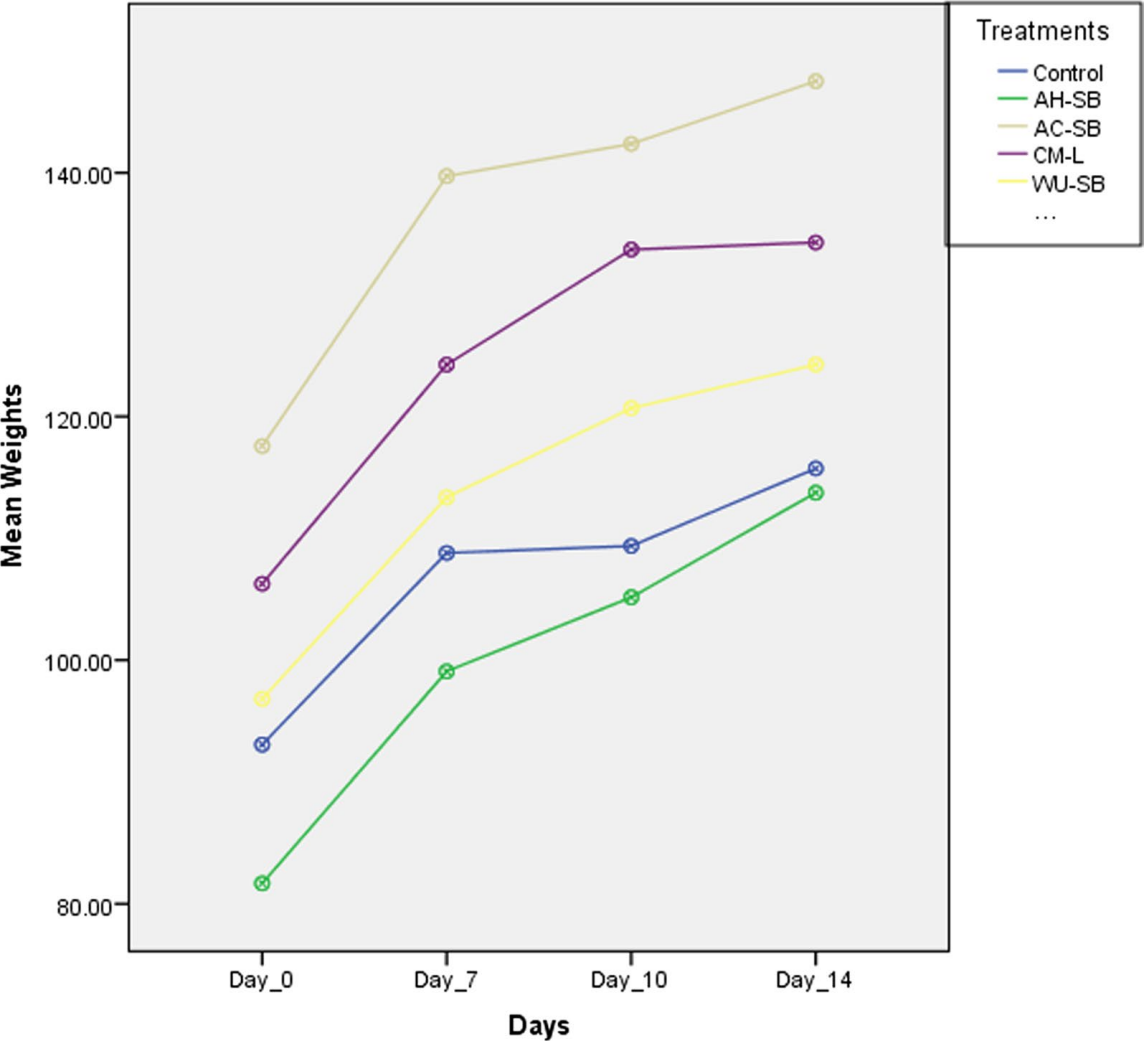
Showing the variation in mean body weights of animals with time

Body weights of animals from both the Control and treated groups increased progressively throughout the experimental period, as shown in Fig. 1. The relative organ weights from the Control and treated groups are presented in Table 5. No significant variations were recorded in the relative organ weights of the treated groups when compared to the Control.

**Table 5:** Effect of extracts of selected plant species on relative weights of organs [(organ weight/body weight) × 100%] of female rats after 14 days

Parameters	Plant species				
	Control	AH-SB	AC-SB	CM-L	WU-SB
Liver	3.92 ± 0.06	3.53 ± 0.37	4.43 ± 0.22	3.55 ± 0.31	3.24 ± 0.13
L-Kidney	0.48 ± 0.03	0.42 ± 0.04	0.47 ± 0.06	0.39 ± 0.04	0.40 ± 0.03
Heart	0.51 ± 0.03	0.52 ± 0.04	0.49 ± 0.00	0.46 ± 0.03	0.51 ± 0.06
Stomach	1.17 ± 0.01	1.55 ± 0.12	1.31 ± 0.04	1.32 ± 0.15	1.16 ± 0.03

*AH-SB* *Acacia hockii* stem bark, *AC-SB* *Albizia coriaria* stem bark, *CM-L* *Combretum molle* leaves, *WU-SB* *Warburgia ugandensis* stem bark. Values are presented as mean ± SEM of triplicates (*n* = 3)

#### Hematological parameters

The results of the hematological analysis are given in Table 6. There were no remarkable alterations in the levels of RBC, HGB, HCT, MCH, PLT, WBC, LYMPH, MONO, and NEUT from all the treated groups compared with the Control group. Nonetheless, the MCHC level increased significantly (*p* < 0.001) in all the treated groups compared to the Control. In addition, a significant decrease was recorded in the level of MCV (*p* < 0.05 and *p* < 0.001) in the *C. molle* and *Z. leprieurii* treated groups, respectively, compared to the Control.

**Table 6:** Hematological parameters (Mean ± SEM) of adult female Wistar albino rats treated with a single dose (2000 mg/kg) of aqueous extracts from selected medicinal plants species after 14 days

Parameters	Plant species					
	Control	AH-SB	AC-SB	CM-L	WU-SB	ZL-RB
RBC (10^6^/µL)	8.40 ± 0.12	8.54 ± 0.36	8.32 ± 0.24	8.64 ± 0.02	8.44 ± 0.18	9.06 ± 0.17
HGB (g/dL)	14.27 ± 0.45	14.80 ± 0.25	14.60 ± 0.40	15.13 ± 0.33	15.30 ± 0.29	14.55 ± 0.25
HCT (%)	53.10 ± 1.53	50.73 ± 1.24	50.53 ± 1.20	49.77 ± 0.90	50.93 ± 0.90	48.45 ± 0.95
MCV (fL)	63.20 ± 1.11	59.50 ± 1.22	60.80 ± 0.32	57.63 ± 0.90*	60.37 ± 1.16	53.45 ± 0.05***
MCH (pg)	16.97 ± 0.32	17.40 ± 0.45	17.57 ± 0.12	17.50 ± 0.35	18.13 ± 0.33	16.05 ± 0.05
MCHC (g/dL)	26.90 ± 0.21	29.17 ± 0.30***	28.90 ± 0.15**	30.40 ± 0.21***	30.03 ± 0.09***	30.05 ± 0.05***
PLT (10^3^/µL)	568.33 ± 35.20	787.33 ± 29.00	887.00 ± 53.50	523.00 ± 220.37	565.67 ± 228.46	858.00 ± 88.00
WBC (10^3^/µL)	4.59 ± 1.53	6.30 ± 0.89	6.36 ± 1.71	5.16 ± 1.12	3.16 ± 1.09	5.26 ± 2.46
LYMPH (%)	67.43 ± 1.82	70.20 ± 3.19	67.13 ± 6.75	83.50 ± 3.31	82.70 ± 1.97	56.75 ± 4.25
MONO (%)	3.40 ± 1.16	0.73 ± 0.15	0.63 ± 0.03	0.40 ± 0.15	0.23 ± 0.12	3.90 ± 2.10
NEUT (%)	24.03 ± 1.67	23.90 ± 2.65	27.33 ± 7.27	14.83 ± 3.48	15.60 ± 1.47	24.40 ± 6.00

*RBC* red blood cells, *HGB* hemoglobin, *HCT* hematocrit, *MCV* mean corpuscular volume, *MCH* mean corpuscular hemoglobin, *MCHC* mean corpuscular hemoglobin concentration, *PLT* platelet, *WBC* white blood cells, *LYMPH* lymphocyte count, *MONO* monocytes, *NEUT* neutrophils. Values are presented as mean ± SEM of triplicates (*n* = 3). *p < 0.05, **p < 0.01, ***p < 0.001 indicate significant changes in comparison with the control

#### Biochemical parameters

The results of the various biochemical tests on the Control and treated groups are summarized in Table 7. Treatment with *A. hockii*, *A. coriaria*, *C. molle*, *W. ugandensis*, and *Z. leprieurii* extracts did not produce statistically significant changes on AST, ALT, ALP, T.P, ALB, and CRE when compared with the Control. However, there was a significant increase in the levels of BILT (*p* < 0.01) and Urea (*p* < 0.05) from the *Z. leprieurii* and *A. hockii* treated groups, respectively, in comparison to the Control.

**Table 7:** Biochemical parameters of adult female Wistar albino rats treated with a single dose (2000 mg/kg) of aqueous extracts from selected medicinal plant species for 14 days

Parameters	Plant species					
	Control	AH-SB	AC-SB	CM-L	WU-SB	ZL-RB
AST (U/L)	261.90 ± 49.97	264.20 ± 43.02	211.23 ± 17.84	271.23 ± 65.38	190.80 ± 9.62	307.25 ± 17.55
ALT (U/L)	62.77 ± 8.20	78.07 ± 2.92	72.70 ± 9.40	57.63 ± 3.79	62.27 ± 6.68	63.45 ± 5.75
ALP (U/L)	80.33 ± 2.03	90.67 ± 10.35	47.33 ± 10.68	90.00 ± 11.93	95.67 ± 6.49	113.00 ± 9.00
TP (g/L)	68.97 ± 2.39	65.73 ± 2.04	73.13 ± 1.65	65.40 ± 0.56	65.27 ± 2.97	69.80 ± 0.60
BILT (μmol/L)	1.40 ± 0.21	0.90 ± 0.12	0.87 ± 0.38	1.30 ± 0.26	1.10 ± 0.12	3.10 ± 0.40**
ALB (g/L)	36.07 ± 1.32	37.90 ± 2.23	46.33 ± 8.49	35.37 ± 0.67	37.53 ± 1.31	49.75 ± 1.75
CRE (μmol/L)	57.33 ± 8.21	39.33 ± 6.69	45.33 ± 4.84	43.00 ± 0.58	46.67 ± 3.71	47.50 ± 1.50
Urea (mmol/L)	7.37 ± 0.32	10.17 ± 0.52*	8.83 ± 0.55	8.73 ± 0.99	8.40 ± 0.26	9.85 ± 0.65

*AST* aspartate aminotransferase, *ALT* alanine aminotransferase, *ALP* alkaline phosphatase, *TP* total proteins, *BILT* bilirubin total, *ALB* albumen, *CRE* creatinine. Values are presented as mean ± SEM of triplicates (*n* = 3). *p < 0.05; **p < 0.01 indicate significant changes in comparison with the control

#### Histopathological analysis

**Fig. 2:**
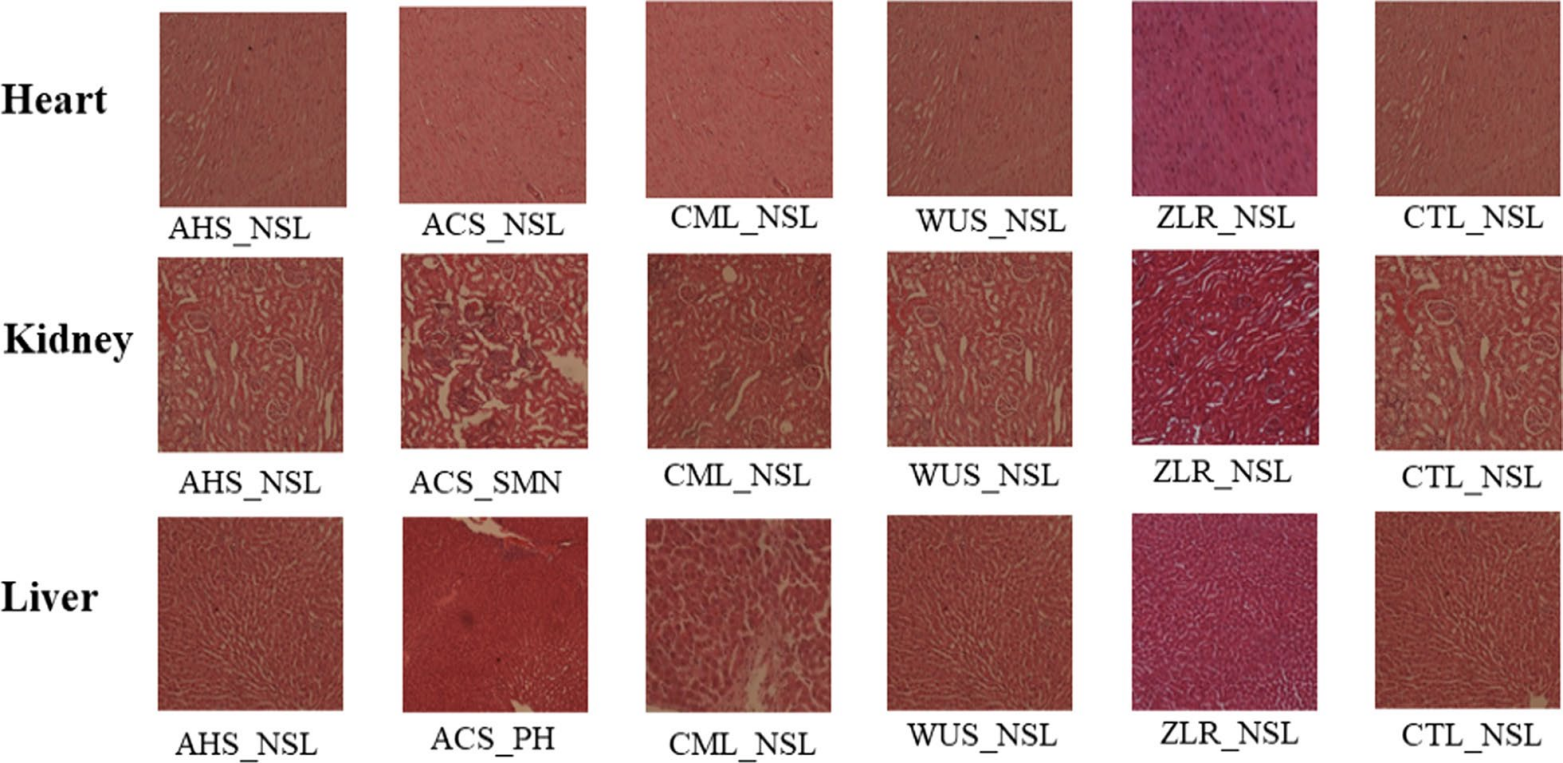
Effects of extracts given at a limit dose (2000 mg/kg body weight) on the histology of the Heart, Kidney, and Liver*.* *AHS* *Acacia hockii*-stem bark, *ACS* *Albizia coriaria*-stem bark, *CML* *Combretum molle*-leaves, *WUS* *Warburgia ugandensis*-stem bark, *ZLR* *Zanthoxylum Leprieurii*-root bark, *CTL* control, *NSL* no significant observable Lesions, *SMN* severe multifocal nephritis, *P.H.* perivascular hepatitis

The histopathological report of the following vital organs: kidney, heart, and liver of animals from the Control and treated groups are presented in Fig. 2, and it suggests that there were no visible significant lesions in the Control and *A. hockii*, *C. molle*, *W. ugandensis*, and *Z. leprieurii* treated groups. However, significant changes in the kidney (severe acute multifocal nephritis) and liver (periportal hepatitis) were observed from the *A. coriaria* treated group.

## Discussion

Phytochemical screening results of the selected extracts revealed the presence of many classes of phytochemical compounds, and it is the first report of the presence of the phytochemicals from the aqueous and methanol/DCM (1:1) extracts of the selected plant species. However, the aqueous and methanol/DCM (1:1) *A. hockii* stem bark extracts from the present study revealed the same phytochemicals as those detected in the *A. hockii* stem bark methanol extract [30]. In addition, all the phytochemicals (except saponins) detected from the *C. molle* leaf extract in the present study are the same as those revealed from the *C. molle* leaf methanol extract [31].

Studies have reported that many classes of phytochemicals have antimycobacterial activity. For example, alkaloids such as ambiguine K, ambiguine A isonitrile, ambiguine C isonitrile, ambiguine E isonitrile, ambiguine I isonitrile, *trans*-fagaramide, hapalindole G, hapalindole H, hydroxy-1, 3-dimethoxy-10-methyl-9-acridone, and solsodomine A, isolated from the berries of *Solanum sodomaeum*, stem bark of *Z. leprieurii* and the Cyanobacterium *Fischerella ambigua*, have antimycobacterial activities [7, 8, 32, 33]. Jujubogenin 3-*O*-α-l-arabinofuranosyl (1 → 2)-[3-*O*-(*trans*)-*p*-coumaroyl-*β*-d-glucopyranosyl (1 → 3)]-α-l-arabinopyranoside, a saponin isolated from *Colubrina retusa*, also showed antimycobacterial activity [34]. Tannins extracted from *C. molle* stem bark such as ellagitannin and punicalagin showed activity against *M. tuberculosis* [35]. In addition, the following flavonoids isolated from various plant species exhibited antimycobacterial activity: amentoflavone, dehydrolupinifolinol, demethoxykanugin, 3,7-dimethoxyflavone, eriosemaone A, flemichin, 4-hydroxylonchocarpin, isobachalcone, kanzanol C, karanjachromene, lacheolatin B, lantanoside, linaroside, lupinifolin, lupinifolinol, maackiain, pinnatin and stipulin [36–38]. β-sitostenone and ergosterol peroxide which are steroids isolated from the twigs and leaves of *R. boniana* showed antitubercular properties [39]. Plant terpenoids including longifolene, sesquiterpene, totarol, and *trans*-communic acid showed activity against *M. tuberculosis* H_37_Rv. Furthermore, totarol and longifolene were active when tested on rifampicin-resistant variants [40]. Therefore, we propose that the antimycobacterial activity exhibited by the selected plant extracts in the present study could be attributed to the main bioactive components, including alkaloids, saponins, tannins, flavonoids, steroids, and terpenoids.

Regarding the isolation of compounds from the selected plant species, no compound has been reported from *A. hockii* stem bark. Meanwhile, acacic acid lactone, lupeol, lupenone, (+)-catechin, betulinic acid, and benzyl alcohol were reported from the ethyl acetate extract of the stem bark of *A. coriaria* [41]. Regarding the chemistry of *C. molle*, steroidal acid saponins were isolated from its leaves [42]. The following sesquiterpenes: cinnamolide, cinnamolide-3β-acetate, linoleic acid, 4(13),7-coloratadien-12,11-olide, 6α, 9α-dihydroxy-4(13),7-coloratadien-11,12-dial, 7β-hydroxy-4(13),8-coloratadien-11,12-olide, mukaadial, muzigadial, muzigadiolide, 11α-hydroxymuzigadiolide, 7α-hydroxy-8-drimen-11,12-olide, ugandensolide, ugandensidial, and warburganal were isolated from the stem bark of *W. ugandensis*. In addition, *Z. leprieurii* methanol root extract afforded the following compounds: 10-O-demethyl-12-O-methylarnottianamide, 10-O-demethyl-12-O-methyl isoarnottianamide, and hesperidin [43].

Both the aqueous and methanol/DCM crude extracts of the selected plant species examined in this study exhibited promising in vitro antimycobacterial activities against the susceptible and MDR strains of *M. tuberculosis*. The activity of the extracts against *M. tuberculosis* was classified based on the following criteria: activity of MIC (μg/mL) < 100 was considered significant, MIC (μg/mL) 100–625 was considered moderate, and weak or low for MIC (μg/mL) > 625 [44].

In general, the methanol/DCM (1:1) extracts registered a higher antimycobacterial activity than aqueous extracts, which agrees with a previous report by Abuzeid et al. (2014). This finding could be attributed to the ability of the medium polar (methanol/DCM, 1:1) solvent system to extract less-polar and lipophilic bioactive molecules which have higher permeability across the lipid cell membranes of the *M. tuberculosis* and thus inhibiting its growth, resulting in higher antimycobacterial activity; meanwhile, the aqueous extracts contain polar molecules with reduced permeability across the membranes leading to low antimycobacterial activity [46]. Many studies have reported less-polar antimycobacterial compounds [47–49]. In addition, the methanol/DCM solvent system, with a wide solvent polarity range, could have afforded a high chemical diversity of the extracted compounds, resulting in synergism in the crude extract and hence, the higher antimycobacterial activity of the extracts. The findings from this study provide solid evidence that the stem bark of *A. hockii*, which showed significant activity, can be a potential source of antimycobacterial molecules. Therefore, it is a good candidate for further scientific investigations to establish the compounds responsible for the activity. The other plant extracts which have exhibited moderate activity are also worth further investigations as they may have highly bioactive compounds, though few. This is the first report of the antimycobacterial activity of the extracts of *A. hockii* (stem bark), *A. coriaria* (stem bark), *C. molle* (leaves), *W. ugandensis* (stem bark), and *Z. leprieurii* (root bark).

Furthermore, the moderate activity of *A. hockii* aqueous extract against the susceptible H_37_Rv could be important, since many previous reports indicated that aqueous extracts lack bioactivity against *M. tuberculosis* [47, 50]. Although this study reports a moderate to low/weak activity exhibited by the aqueous extracts, consuming a significant amount of the extracts could treat tuberculosis. It may help explain the therapeutic effects of the selected plant species as claimed by the local communities.

Further still, since the bactericidal activity is defined by the ratio of MBC to MIC being less than or equal to 4, methanol/DCM (1:1) extracts of *A. hockii*, *A. coriaria*, *C. molle,* and *W. ugandensis* are bactericidal, while that of *Z. leprieurii* is bacteriostatic [51]. This is particularly important because bactericidal anti-TB agents are needed to avoid relapse and lessen the risk of developing resistance in *M. tuberculosis* [52].

From previous studies, the following ethnobotanical uses apart from antimycobacterial activity have been reported for the plant species in this study. *A. hockii* has antioxidant and anti-inflammatory activities [53, 54]. The root or stem barks of *A. coriaria* are used as a general tonic and to treat syphilis, cough, skin diseases, eye diseases, jaundice, and sore throat [14, 55]. It is also used to treat diarrhea, stomach ache, cancer, malaria, antibacterial and antifungal infections, and dermatological diseases [56, 57]. *C. molle* is used as a remedy for several diseases, including aphrodisiac, malaria, pain, bacterial infections, ulcers, diarrhea, hemorrhoids, syphilis, and also to manage HIV/AIDS-related symptoms [58, 59]. The leaves, root, and stem bark of *W. ugandensis* are used locally to treat the following conditions: allergies, anemia/blood infections, candidiasis, cough/T.B/asthma, diarrhea, fallopian tube blockage, fatigue, fever, influenza, hypertension, measles, miscarriage, nasal congestion, nose bleeding, sore throat, stomach ache, syphilis, ulcers, skin rashes and also, to manage HIV/AIDS [14, 59]. *Z. leprieurii* is used to treat urinary infections, rheumatic pain, and malaria. It is also used as an antiseptic and manages HIV/AIDS-related symptoms [60].

On the other hand, toxicological assessment is crucial for authentication of the safety of herbal medications. There are no previous reports about the acute and subacute toxicity studies of the plants selected in the present study. Therefore, the current study was also conducted to assess the acute toxicity profiles of aqueous extracts of the selected plant species in animal models using the OECD guidelines 425 [27]. This is because an acute toxicity study is needed to establish a safer dose range to manage the clinical signs and symptoms of the drugs [61]. Consequently, according to Namulindwa et al. (2015), the excessive urination recorded from the *A. coriaria* and *A. hockii* treated groups in this study could be linked to toxicity. Furthermore, no significant changes were detected in body weight gains of animals in the treated groups during the study, and this suggests that the normal processing of all the nutrients such as carbohydrates, fats, and proteins have been metabolized appropriately within the body as these nutrients play a significant role in physiological functions [63].

Vital organs, including the liver, heart, kidney, spleen, and lungs, are functionally crucial organs often impaired by toxic substances [64]. In this study, the Control, and treated groups, *A. hockii*, *C. molle*, *W. ugandensis,* and *Z. leprieurii* showed no visible significant lesions upon histological examinations and, therefore, may not be acutely toxic to the vital organs. However, the *A. coriaria* treated group’s findings showed clear evidence of organ toxicity, where severe multifocal nephritis and perivascular hepatitis, more pronounced in the portal area, were observed in the kidney and liver, respectively. The severe multifocal nephritis observed in this study is most likely linked to an *A. coriaria* extract-induced nephritis characterized by inflammatory cells infiltration at various locations in the renal interstitium. Furthermore, perivascular hepatitis in the liver could be linked to an *A. coriaria* aqueous extract induced hepatocellular injury leading to the observed inflammatory cells (mainly neutrophils and lymphocytes) aggregated around the blood vessels, particularly in the hepatic portal area. These findings corroborate well with the clinical signs of toxicity, such as increased respiration, excessive urination, and defecation, and the single mortality registered upon oral administration of *A. coriaria* extract. These findings also agree with many studies which have reported toxic extracts/compounds from the Genus Albizia [65–68]. This indicates that *A. coriaria* is moderately toxic at the current dose of 2000 mg/kg.

Hematological parameters are sensitive biomarkers of the physiological changes in reaction to any toxic substances [69]. In this study, there was a significant decrease in the level of MCV from the *C. molle* and *Z. leprieurii* treated groups. Since MCV measures the average size or volume of RBC, a low MCV (microcytic) observed in the *C. molle* and *Z. leprieurii* treated groups is consistent with anemia [70–72]. From the results obtained, it may be argued that aqueous extracts of *C. molle* (leaves) and *Z. leprieurii* (root bark) may cause anemia.

Numerous toxic compounds accumulate in the liver, where they are detoxified [73]. Liver damage is typically assessed by determining serum transaminases (AST and ALT) and measuring total proteins. Liver injury caused by hepatotoxic drugs can result in elevated AST, ALT, and total protein levels [74, 75]. Hepatocellular damage could increase cell membrane permeability and then lead to the release of aminotransferases into the bloodstream [76, 77]. Since no significant alterations were detected in serum levels of these three markers of liver function, and histopathological analyses of the livers from treated animals did not show tissue changes, the findings from the present study suggest that administration of the extracts from the selected plant species did not cause liver damage.

The kidneys receive about 25% of the cardiac blood flow, and any substance that reaches the systemic circulation will reach this organ; hence, they are considered frequent toxicity targets [78]. Renal function is usually assessed by serum creatinine and urea levels and histopathological analysis of the kidney tissues. Its impairment is shown by raised serum creatinine levels and urea [79]. Urea is a marker of acute renal dysfunction, the first acute marker following renal injury [80]. In this study, a significant increase in the level of urea was observed in the *A. hockii* treated group, suggesting a mild renal injury. This finding is also supported by the excessive urination observed from the same group.

According to the OECD guidelines No 425 [27], the LD 50 of a substance is considered to be greater than 2000 mg/kg if three or more experimental rats in a group of five survive after administering a limit dose of 2000 mg/kg body weight. In this study, mortality was registered from the *A. coriaria* treated group only, where one rat died. This places the LD 50 of all the groups at greater than 2000 mg/kg. Therefore, since chemicals are divided into five groups based on their LD50 according to the globally harmonized classification system [81], all the extracts considered in this study can be classified into group 5 (LD50 > 2000 mg/kg), which is the class of least toxicity. In spite of this general classification, signs of toxicity were detected in the organs from the *A. coriaria* treated group and could be considered somewhat toxic. This is the first report of acute toxicity study of all the selected plant species to the best of our knowledge.

## Conclusions

Phytochemical screening of the extracts revealed the presence of alkaloids, tannins, saponins, flavonoids, steroids, terpenoids, resins, cardiac glycosides, phenolic compounds, and coumarins. The selected medicinal plants have promising antimycobacterial activity, and low toxicity, except *A. coriaria,* which appears to be moderately toxic. The findings of this study justify the ethnopharmacological use of the plant parts of the selected plant species to treat tuberculosis and other diseases whose symptoms closely resemble tuberculosis. Chronic toxicity studies of the selected plant species will be necessary to support their use further. Furthermore, all the selected plant species in this study are potential candidates for isolation and characterization to identify antimycobacterial compounds responsible for their activities.

## Supplementary Information

**Additional file 1:** The detailed descriptions of the standard methods for preliminary phytochemical analysis, the microplate Alamar blue assay (MABA) protocol, and Figure S1 showing the observed efficacy of the extracts using the susceptible Mycobacterium tuberculosis strain (H_37_Rv).

## Data Availability

All data generated or analyzed during this study are included in this published article.

## Abbreviations

AIDS: Acquired immunodeficiency syndrome
ANOVA: Analysis of variance
DMSO: Dimethyl sulfoxide
HIV: Human immunodeficiency virus
LD: Lethal dose
