# Supplementary material for: Phytochemical screening, antimycobacterial activity and acute toxicity of crude extracts of selected medicinal plant species used locally in the treatment of tuberculosis in Uganda
Source: Trop Med Health. 2022 Feb 17;50:16. doi: 10.1186/s41182-022-00406-7 (PMC8851836; doi:10.1186/s41182-022-00406-7)
Supplement: Supplementary file 1 — Additional file 1: The detailed descriptions of the standard methods for preliminary phytochemical analysis, the microplate Alamar blue assay (MABA) protocol, and Figure S1 showing the observed efficacy of the extracts using the susceptible Mycobacterium tuberculosis strain (H37Rv). [file 41182_2022_406_MOESM1_ESM.pdf]

Supplemental Material

Phytochemical Screening, Antimycobacterial activity and Acute toxicity of crude extracts of selected medicinal plant species used locally in the treatment of Tuberculosis in Uganda

Benson Oloya[1,2*], Jane Namukobe[1], Willy Ssengooba[3], Mathias Afayoa[4], Robert Byamukama[1]

Corresponding author

Benson Oloya, Department of Chemistry, College of Natural Sciences, Makerere University P.O. Box 7062, Kampala-Uganda. b.oloya@muni.ac.ug

Abstract

Background: Tuberculosis (TB) is one of the leading causes of death globally, and the rise in drug-resistant forms of TB has become a significant threat. Subsequently, it is crucial to explore new, effective and safe anti-TB agents. This study aimed at conducting phytochemical screening, antimycobacterial activity, and acute toxicity of the selected plant species' crude extracts to assess their toxicological potentials and efficacies against TB.

Methods: The aqueous and methanol/dichloromethane (DCM) (1:1) extracts of each selected plant species were subjected to phytochemical screening and antimycobacterial activity using microplate alamar blue assay. For acute toxicity, a single dose (2000 mg/kg) of the aqueous extracts was orally administered to each animal following the Organization for Economic Cooperation and Development (OECD) guidelines No. 425 and then observed for 14 days. The animals were closely observed on the general behavior and clinical signs of toxicity, and body weights were recorded. After the termination of the experiment, hematological, biochemical, and histopathological analyses were performed.

Results: The extracts contained alkaloids, flavonoids, tannins, saponins, steroids, terpenoids, resins, cardiac glycosides, phenolic compounds, and coumarins. Aqueous extracts showed moderate to weak activity against the susceptible ($H_{37}Rv$) *M. tuberculosis* strain and weak activity against the MDR-TB

strain with Minimum Inhibitory Concentrations (MIC μg/mL) ranging from 293.0-2344.0 and 1172.0-4688.0, respectively. Methanol/DCM extracts showed significant to moderate activity against the susceptible TB strain and moderate to weak activity against the MDR-TB strain with MIC (μg/mL) ranging from 98.0-586.0 and 293.0-781.0, respectively. One mortality was recorded from the *A. coriaria* treated group following the acute toxicity tests, but the LD_{50} of all the extracts was estimated to be above 2000 mg/kg. Histopathological analyses did not show any significant lesions in the examined organs except those from the *A. coriaria* treated group.

Conclusion: Phytochemical screening of the extracts revealed the presence of alkaloids, tannins, saponins, flavonoids, steroids, terpenoids, resins, cardiac glycosides, phenolic compounds, and coumarins. All the methanol/DCM extracts of the plant species studied have promising antimycobacterial activity. The selected plant extracts studied exhibited low acute toxicity levels except for *A. coriaria* and could be safe for formulations into herbal products.

Keywords: Phytochemistry, Antimycobacterial activity, Acute Toxicity, Tuberculosis, Medicinal plants.

Materials and methods

1.1 Phytochemical screening of extracts

Both the aqueous and methanol/DCM (1:1) extracts from each of the selected plant species were analyzed for alkaloids, saponins, tannins, flavonoids, steroids, cardiac glycosides, resins, anthraquinones, phenolic compounds, phlobatannins, coumarins, and terpenoids. Standard methods for preliminary phytochemical analysis were used with some minor modifications [1, 2].

1.1.1 Test for Alkaloids

Wagner's test. The extract (1 ml) was mixed carefully with freshly prepared Wagner's reagent (3 drops). The formation of brown precipitates indicated the presence of alkaloids in the extract.

1.1.2 Test for Saponins

Froth test. The extract (0.5 g) was dissolved in distilled water (10 ml). The solution was covered and shaken for 30 s. Then it was allowed to stand for 30 min. The formation of honeycomb froth above the surface after 30 min showed the presence of saponins.

1.1.3 Test for Tannins

(i) *Braymer's test*. The extract (0.2g) was stirred with distilled water and filtered. 10% Ferric chloride (3 drops) was added to the filtrate. A blue-black, green or blue-green precipitate was taken as evidence for the presence of tannins.

1.1.4 Test for Flavonoids.

(i) A small amount of magnesium powder and few drops of concentrated hydrochloric acid were added to the extract (3 ml). A red or intense red coloration indicated the presence of flavanones.

(ii) *Reaction with sodium hydroxide*. To the extract (3 ml), dilute NaOH solution (2 ml) was added. The formation of intense yellow color, which becomes colorless on the addition of dilute hydrochloric acid, indicated the presence of flavonoids.

1.1.5 Test for Steroids

Salkowski's test. The extract (0.2 g) was dissolved in chloroform (2 ml). Concentrated sulphuric acid was carefully added to form a lower layer. A reddish-brown color at the interphase indicated the presence of steriods.

1.1.6 Test for Cardiac Glycoside

Keller-Killani Test. The extract (0.2 g) was dissolved in glacial acetic acid (2 ml) containing one drop of 5 % ferric chloride solution. Carefully, few drops of concentrated sulphuric acid were

added to the sides of the test tube. A brown ring at the interface confirmed the presence of a cardiac glycoside.

1.1.7 Test for Resins

Copper acetate solution (5 ml) was added to the extract (5 ml). The resulting solution was shaken vigorously and allowed to separate. A green-colored solution was taken as evidence of the presence of a resin.

1.1.8 Test for Anthraquinones.

Borntrager's test. The extract (0.2 g) was shaken with 4 ml of benzene. The mixture was filtered, and 10% ammonia solution (2 ml) was added to the filtrate. The mixture was shaken, and the presence of pink, red, or violet color in the ammoniacal (Lower) phase indicated the presence of free anthraquinones.

1.1.9 Test for Phenolic compounds

Ferric chloride test. The extract (0.2 g) was dissolved in a 5% Ferric chloride solution. A green or dirty green precipitate indicated the presence of a phenolic compound.

1.1.10 Test for Phlobatannins

The extract (0.5 g) was dissolved in distilled water and filtered. The filtrate (2 ml) was boiled with 1% HCl (2 ml) solution. Red precipitate showed the presence of Phlobatannins.

1.1.11 Coumarins

(i) *Reaction with alcoholic NaOH*. The extract (2 ml) was mixed with few drops of alcoholic NaOH solution. The appearance of yellow color indicated the presence of coumarins.

(ii) *NaOH test*. To the plant extract was added 10% NaOH followed by chloroform. The appearance of yellow color indicated the presence of coumarins.

1.1.12 Terpenoids

The extract (1 g) was placed in a test tube, then methanol (10 ml) was poured on it, shaken well, and filtered to make 5 ml of extract of plant sample. Then chloroform (2 ml) was mixed with the extract, and concentrated sulphuric acid (3 ml) was added. The formation of reddish-brown color indicated the presence of terpenoids in the extract.

1.2 Determination of Minimum Inhibitory Concentration (MIC) and Minimum Bactericidal Concentration (MBC)

The MIC and MBC of the crude extracts against both the susceptible ($H_{37}Rv$) and MDR-TB strains were determined using microplate alamar blue assay (MABA) protocol with minor modifications [3]. All the outer perimeter wells of the sterile 96-well plates were filled with sterile distilled water (200 µl) to minimize evaporation. To each of the remaining wells, 100 µL of 7H9 broth was added. To each of the wells in rows B to G in column 2, double concentration drug solutions (100 µL) were added. Then, using a multi-channel pipette, two-fold serial dilutions were made through column 10. All the wells in columns 2–11 were inoculated with 100 µl of *M. tuberculosis,* bringing the final volume to 200 µl per well. The plates were incubated at 37°C for 21 days. Freshly prepared alamar blue reagent (30 µL) was later added to one of the control wells and incubated at 37°C for 24 h. Observation of a color change indicated that there was growth. Later, the dye was added to all the wells and incubated for 24 h. Standard drugs: isoniazid and rifampicin were used as a positive control and drug-free wells as a negative control. The tests were prepared in triplicates for each of the strains used and performed in a Mycobacteriology Laboratory (BSL-3) at the College of Health Sciences, Makerere University.

Results

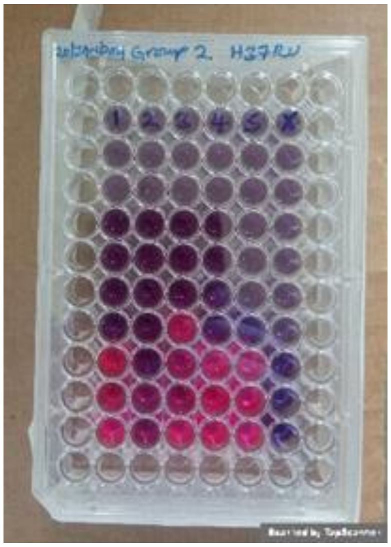

Figure S1: Showing the observed efficacies of the extracts using the laboratory control strain (H37RV). Purple shows there was no growth while pink shows growth.

References

1. Shaikh JR, Patil M. Qualitative tests for preliminary phytochemical screening: An overview. Int J Chem Stud. 2020;8:603–8. doi:10.22271/chemi.2020.v8.i2i.8834.

2. Singh V, Kumar R. Study of Phytochemical Analysis and Antioxidant Activity of *Allium sativum* of Bundelkhand Region. Int J Life-Sciences Sci Res. 2017;3:1451–8. doi:10.21276/ijlssr.2017.3.6.4.

3. Lawal TO, Adeniyi BA, Wan B, Franzblau SG, Mahady. In Vitro Susceptibility of Mycobacterium tuberculosis to extracts of Uvaria afzelli and Tetracera alnifolia. African J Biomed Res. 2011;14:17–21.
